# How Financial Incentives Increase Smoking Cessation: A Two-Level Path Analysis

**DOI:** 10.1093/ntr/ntaa024

**Published:** 2020-01-29

**Authors:** Floor A van den Brand, Math J J M Candel, Gera E Nagelhout, Bjorn Winkens, Constant P van Schayck

**Affiliations:** 1 Department of Family Medicine, Maastricht University (CAPHRI), Maastricht, The Netherlands; 2 Department of Health Promotion, Maastricht University (CAPHRI), Maastricht, The Netherlands; 3 IVO Research Institute, The Hague, The Netherlands; 4 Department of Methodology and Statistics, Maastricht University (CAPHRI), Maastricht, The Netherlands

## Abstract

**Introduction:**

Financial incentives effectively increase smoking cessation rates, but it is unclear via which psychological mechanisms incentives influence quit behavior. The current study examines how receiving financial incentives for smoking cessation leads to quitting smoking and investigates several mediators and moderators of that relationship.

**Aims and Methods:**

The study sample consisted of 604 tobacco-smoking employees from 61 companies in the Netherlands who completed a baseline and follow-up questionnaire. The current study is a secondary analysis from a cluster randomized trial where employees received smoking cessation group counseling at the workplace. Participants in the intervention group additionally received financial incentives of €350 in total for 12-month continuous smoking abstinence. We used a two-level path analysis to test a model that assesses the effects of financial incentives through smoking cessation program evaluation, medication use, nicotine replacement use, attitudes, self-efficacy, and social influences on quit success. We additionally tested whether an individual’s reward responsiveness moderated the influence of incentives on quit success.

**Results:**

The effect of financial incentives on quit success was mediated by a higher self-efficacy. Financial incentives were also associated with a higher use of cessation medication. A more positive program evaluation was related to higher self-efficacy, more social influence to quit, and more positive attitudes about quitting. The results did not differ significantly by individual reward responsiveness.

**Conclusions:**

The results of the current study suggest that financial incentives may be used to increase medication use and self-efficacy for quitting smoking, which offers an indirect way to increase successful smoking cessation.

**Implications:**

(1) This is the first study investigating via which psychological pathways financial incentives for quitting smoking can lead to long-term quit success. (2) The results showed a path between financial incentives and a higher likelihood of medication use. Incentives may encourage smokers to use medication in order to increase their chance of quitting smoking and receive the reward. (3) There was a path from financial incentives to quit success via a higher self-efficacy. (4) The effects of financial incentives did not depend on individual reward responsiveness.

## Introduction

Financial incentives are used to promote health-related behavior or to motivate behavior change in different fields, including smoking cessation.^1–4^ Although financial incentives for smoking cessation have shown to increase quit rates in a growing body of research, it is not exactly known how incentives work. It has been suggested that incentives are effective because they provide an attainable goal in the near future^5^ and because providing financial incentives is a form of operant conditioning that can lead to voluntary changes in habitual behavior.^6^ However, it is not clear via which psychological mechanisms financial incentives influence quit behavior, and via which pathways incentives lead to an increase in smoking cessation success.^7–10^ A better understanding of the workings of financial incentives can help to design effective interventions to reduce smoking prevalence, as it can provide insight into which other elements within an intervention with financial incentives can be adapted to improve the intervention’s effect. For example, financial incentives may, apart from increasing quit success directly, have a positive effect on other factors that are known to increase quit success, such as a higher willingness to use smoking cessation medications or nicotine replacement therapy (NRT). This could subsequently be a reason to make these medications easily accessible together with an intervention with financial incentives.^11^ In a previous study, we performed a cluster randomized trial (CRT) where employees participated in a smoking cessation group training organized at the workplace.^9,12^ Participants in the intervention group earned vouchers if they succeeded at quitting smoking, which resulted in significantly higher quit rates after 12 months in the intervention group compared with the control group.^9^ In the current study, we will test a model of the psychological pathways leading from financial incentives to quit success via smoking cessation training program evaluation, smoking cessation medication use, NRT use, and the three psychosocial mediators attitude, social influence, and self-efficacy, known from several behavioral change theories such as the ASE (Attitude, Social influence, Efficacy)-model^13^ and Theory of Planned Behavior.^14^

Financial incentives could increase the probability that a person successfully quits smoking by enhancing motivation to quit,^15^ but could also work through different mediators, for example if incentives stimulate smokers to make use of a smoking cessation treatment.^11^ In a CRT where incentives for quit success were offered without any cessation support, participants in the incentives group reported more often that they obtained help with quitting smoking on the internet.^16^ In a different study, incentives stimulated people to call a quit line more often.^17^ Additionally, a qualitative study among pregnant smokers based in the United Kingdom found that pregnant women appeared to have used the National Health Service (NHS) Smoking Services more because of incentives and suggested that the positive affect created by the incentives may have caused the women to regard the Smoking Services more positively.^18^ In the current study, we test the idea that because of financial incentives, people may be more positive toward participating in a smoking cessation training program, which may increase their engagement with the program and could, in turn, enhance its effectiveness. Likewise, we examine whether financial incentives for quit success may make people more inclined to use smoking cessation medications to improve their chances of quitting smoking, which can indirectly lead to higher success rates.^19^

The effect of financial incentives on quit success may furthermore run through the psychosocial mediators attitudes, social influences, and self-efficacy, which predict a person’s intention to change their behavior and can lead to behavioral change such as quitting smoking.^13,14,20,21^ To our best knowledge, the current study is the first to examine whether these psychosocial factors mediate the effect of financial incentives on smoking cessation, which leaves us to speculate about possible mechanisms. For example, participants may like the idea of being able to earn rewards for quitting smoking and enjoy receiving the rewards,^15,22^ which may contribute to a positive attitude toward quitting smoking. Social influences from a smoker’s environment include the subjective norm toward smoking, the perceived social pressure to quit smoking, and social support for quitting smoking.^20^ When financial incentives are provided for quitting smoking this may propagate a social norm of nonsmoking,^20^ and smokers may receive more encouragement from people in their social environment to reach their goal of quitting smoking and receiving the reward, for example when a spouse also profits from the reward.^23^ Finally, financial incentives may increase a person’s self-efficacy to quit smoking, for example if they stimulate visualization of success.^24^

The effect of financial incentives on motivating behavior change may differ among individuals.^15^ It may be influenced by how attractive a specific incentive is to an individual.^22^ The effect of incentives could also depend on how sensitive a person is to rewards in general.^25^ Greater perceived importance of receiving incentives for abstinence has been associated with increased smoking cessation success in a previous study.^26^ The effect of financial incentives on quit success may therefore be stronger for individuals who are more responsive to rewards.^25,27^

The current study examines how receiving financial incentives for smoking cessation leads to quitting smoking and investigates several mediators of that relationship. We hypothesize that financial incentives influence smoking cessation via a more positive appraisal of the smoking cessation training program, higher medication, and NRT use and through a positive effect on the psychosocial mediators attitudes, self-efficacy, and social influence. Finally, we hypothesize that these associations are dependent on an individual’s responsiveness to rewards.

## Materials and Methods

### Design

The data used for this study are from a CRT performed within Dutch companies, of which the methods and results have been described in more detail in previous publications.^9,12^ In the CRT, 604 employees from 61 companies received the same group smoking cessation training program at their workplace which consisted of seven weekly sessions of 90 min. The group training program was provided by the Dutch company SineFuma. The training program has various components that help participants to quit smoking, and includes enhancing motivation to quit smoking, creating positive attitudes for quitting, and improving self-efficacy for quitting smoking and maintaining abstinence. The group component stimulates social support, peer pressure, and a positive social norm toward quitting smoking. Finally, the program increases knowledge about nicotine addiction, the positive effects of quitting smoking, and provides information on smoking cessation medications. After cluster randomization, 31 companies (320 employees) were allocated to the intervention group and 30 companies (284 employees) to the control group. Employees from companies in the intervention group earned vouchers of €350 in total if they had quit smoking successfully. All employees were aware of the incentives before the start of the program, but the result of the randomization was unknown to participants up until it was revealed during the first training session. Data were collected between March 2016 and March 2018 via online questionnaires, which were distributed at baseline, at the first follow-up measurement which was directly after finishing the training program, and at three months, six months and 12 months after the training program. The study was registered in the Netherlands Trial Register (NL5537) and cleared by the medical-ethical committee METC Z in Heerlen, The Netherlands.

### Participants

All participants from the CRT were included in the current study (*n* = 604). Participants were employees from Dutch companies of at least 18 years old who smoked tobacco. There were no exclusion criteria regarding the amount of tobacco that the participants smoked.

### Measurements

#### Control Variables

Control variables were gender, age, highest completed educational level, income level, and nicotine dependence. The highest completed educational level was divided into three categories: low (none completed, primary school, lower secondary education), moderate (middle secondary education), and high (upper secondary education, university). Income was based on individualized net household income and divided into three groups based on tertiles (€0–€1630, €1630–€2210, €2210, and higher). Nicotine dependence was measured using the Fagerström score ranging from 0 to 10 where higher scores indicate a higher nicotine dependence.^28^

#### Financial Incentives

Participants in the intervention group received vouchers for continuous abstinence from smoking, validated by expired-air carbon monoxide (CO) measurement. CO measurements were performed and vouchers were distributed upon abstinence directly after finishing the smoking cessation program (€50), after three months (€50), after six months (€50), and after 12 months (€200). Participants in the control group did not receive incentives. The variable financial incentives was categorized as “incentives group” (1) and “control group” (0).

#### Smoking Cessation Group Training Program

As a measure for appraisal of the group training program, we assessed how participants evaluated the program using 13 questions (see Supplementary File 1) that were specifically designed for the current study, which each could be answered on a 5-point Likert scale from 1 (completely disagree) to 5 (completely agree). The questions concerned the quality, content, and duration of the training, the communication with the trainer, and whether participants liked and were satisfied with the program and would recommend it to others. Additionally, we asked the participants to grade the training with a score ranging from 1 (worst) to 10 (best). The scores on the evaluation items were averaged into a single scale score, where the grade score was first rescaled into a range from 1 to 5. Cronbach’s alpha was 0.86.

#### Medication and Nicotine Replacement Therapy

Participants could fill out whether they had used smoking cessation medication or NRT during their quit attempt. Both medication use and NRT were coded as “yes” (1) or “no” (0), indicating whether the participant used at least one medication or NRT for smoking cessation.

#### Psychosocial Mediators

Attitudes about smoking cessation were measured with the question: “If you quit smoking within the next 3 months, this would be…” for participants who were smokers, and with the question “If you stay abstinent from smoking for the next 3 months, this would be…” for participants who had quit smoking. Participants could answer on three 5-point scales if they thought this would be wise or unwise, pleasant or unpleasant, and positive or negative.^20^ Cronbach’s alpha was 0.67. The scale score, being the average of the item scores, was used for the analysis. Self-efficacy for quitting smoking was assessed by asking: “Suppose you want to quit smoking within the next 3 months (first part was presented only to smokers), will you be able to resist smoking when: (1) you just woke up? (2) you have experienced something annoying? (3) you are having a cup of coffee or tea? (4) you are drinking alcohol? (5) you are offered a cigarette?” ^29^ Response options ranged on a 5-point scale from “I will certainly not be able” (1) to “I will certainly be able” (5). Cronbach’s alpha was 0.79. The average of the item scores was the scale score and was used in the analysis.

Social influence was measured by the subjective norm toward quitting smoking and social support for quitting smoking. Subjective norm was measured with the question: “How do you think that people who are important to you would feel if you did not smoke in the next three months?” Participants could answer this question on a 5-point scale from “strongly disapprove” (1) to “strongly approve” (5).^20^ Social support that participants received from (a) their partner, (b) friends and family, (c) colleagues who also participated in the group smoking cessation program, and (d) colleagues who did not participate in the cessation program was assessed with the question: “How supportive do you think your [a–d] would be if you attempted to quit smoking?” Response options were “very supportive” (3), “moderately supportive” (2), “little supportive” (1), and “don’t know.” The “don’t know” category was classified as missing. Cronbach’s alpha was 0.62. The scale score, being the average of the item scores, was used for the analysis.

#### Reward Responsiveness

Reward responsiveness was measured using the Behavior Activation System (BAS) Reward Responsiveness Scale consisting of five statements that participants could agree or disagree with,^30^ for example: “When I get something I want, I feel excited and energized.” Response options consisted of a 4-point scale ranging from “very false for me” (1) to “very true for me” (4). A higher score on an item indicates a higher reward responsiveness. Cronbach’s alpha was 0.75. The average of the item scores yielded the scale score, which was used in the analysis.

#### Quit Success

Quit success was defined as CO validated continuous smoking abstinence 12 months after finishing the training program. A research assistant made appointments to visit participants at the workplace who reported to be abstinent from smoking to biochemically validate smoking abstinence using CO measurement. Over the 12-month follow-up period, CO measurements were performed directly after finishing the smoking cessation program, and three, six and 12 months after finishing the program. If CO validation could not be performed, for example due to an employee’s illness or vacation, or if the CO measurement exceeded the threshold of 9 parts per million (ppm),^31^ the participant was considered to be a smoker.

### Statistical Analyses

IBM SPSS Statistics for Windows (version 25.0, Armonk, NY: IBM Corp) was used for analyses of sample characteristics, loss to follow up, reliability of scales, and correlations. Mplus version 7.3 was used to perform the two-level path analysis. We assessed model fit using the comparative fit index, the Tucker–Lewis index, and the root-mean-square error of approximation. We consider the model to be a good fit if the comparative fit index and Tucker–Lewis index were greater than 0.90 and the root-mean-square error of approximation was less than 0.05.^32^

We first tested a model of the effect of financial incentives for smoking cessation via both a direct pathway on 12-month smoking abstinence, and via the psychosocial mediators attitudes, self-efficacy, and social influence, group training evaluation, medication use, and NRT use. We declared a path between financial incentives and quit success statistically significant if all intermediate associations of a path were also significant (Joint Significance Test).^33^ We tested a second model to examine whether reward responsiveness moderated the effect of incentives. We adjusted the analyses for the first and the second model for all control variables mentioned in the Measurements section. For the second model this also involved including interactions between financial incentives and these control variables. Attitudes, self-efficacy, social influence, group training evaluation as well as reward responsiveness were included via means of the associated item scores in the analysis. We used the full conditional specification method (with the sequential regression procedure) to impute missing data. Several simulation studies suggest that this imputation method produces unbiased parameter estimates and standard errors.^34,35^ The number of imputations was 55 and was set at least as large as the percentage of incomplete cases.^36^ As predictors for the multiple imputation we used all variables in the path analysis model. We also performed a complete case analysis including only participants with no missing data as a sensitivity analysis. In both the imputation and analysis phase (for available and complete cases), care was taken of possible clustering effects due to persons being nested within companies, by inclusion of a random intercept at the company level for each dependent variable in the model. Since medication use, NRT, and abstinence at 12 months were binary variables, robust maximum likelihood was used, involving sandwich estimators of the standard errors of the regression coefficients.

## Results

### Loss to Follow-up

Of the 604 participants included in the CRT, 62 (9.9%) did not fill out the follow-up questionnaire that was administered directly after finishing the smoking cessation program. The participants who did not complete the questionnaire directly after finishing the program had more often a low income level (≤€1630) than participants who did complete the questionnaire (58% vs. 31%, *p* = .001), had a higher mean nicotine dependence (Fagerström score 5.2 vs. 4.3, *p* = .004), but did not depend significantly on education level, age, sex, and intervention condition.

### Participants

Participant characteristics are presented in Table 1. Participants had a high reward responsiveness with a mean score of 3.6 for a maximum of 4. Attitudes about quitting were positive on average, just like the perceived social norm toward quitting and social support. Self-efficacy of participants was high with a mean score of 4.5 out of 5.0. Participants graded the smoking cessation group training program with a high mark of 7.8 out of 10. Of the total number of 604 participants in the study, 34% (206/604) had a CO value less than or equal to 9 ppm and were registered as continuously abstinent between the end of the smoking cessation program and 12 months later. In the intervention group with financial incentives the 12-month quit rate was 41% (131/319) and in the control group 26% (75/285).

**Table 1.** Participant Characteristics at Baseline, After Finishing the Smoking Cessation Group Training Program and After 12 Months (*n* = 604)

Characteristic	Baseline	After the smoking cessation training program	After 12 months
Age (*n* = 599)	45.1 (10.2)	—	—
Sex (*n* = 604)		—	—
Women	223 (36.9%)		
Men	381 (63.1%)		
Educational level (*n* = 579)		—	—
Low	159 (27.5%)		
Moderate	255 (44.0%)		
High	165 (28.5%)		
Income level (*n* = 485)		—	—
Low	179 (33.5%)		
Moderate	175 (32.7%)		
High	181 (33.8%)		
Nicotine dependence (Fagerström score 1–10; *n* = 573)	4.4 (2.0)	—	—
Reward responsiveness^a^ (*n* = 580)	3.6 (0.4)	—	—
Financial incentives (*n* = 604)		—	—
Yes	319 (52.8%)		
No	285 (47.2%)		
Medication use (*n* = 536)			
Yes	—	120 (22.4%)	—
No		416 (77.6%)	
Nicotine replacement therapy (*n* = 537)			
Yes	—	264 (49.2%)	
No		273 (50.8%)	
Attitudes about quitting (1–5) (*n* = 524)	—	4.7 (0.5)	—
Self-efficacy for quitting (1–5) (*n* = 522)	—	4.5 (0.5)	—
Social support for quitting (1–3) (*n* = 520)	—	2.4 (0.5)	—
Subjective norm about quitting (1–5) (*n* = 523)	—	4.7 (0.5)	—
Training program evaluation (1–5) (*n* = 517)	—	3.9 (0.7)	—
Training program score (1–10) (*n* = 512)	—	7.8 (1.4)	—
Carbon monoxide scores of participants abstinent at 12 months follow-up (*n* = 206)			
≤4 ppm			199 (96.6%)
≤9 ppm			206 (100.0%)
Quit success (*n* = 603)			
Yes	—	—	206 (34.2%)
No			397 (65.8%)

Numbers are mean (SD) or *n* (%).

^a^BAS Reward Responsiveness Scale. A higher score on the scale (maximum 4) indicates a higher reward responsiveness.

### Correlations

Table 2 displays correlations between financial incentives, training program, medication use, NRT use, reward responsiveness, psychosocial mediators, and successful quitting smoking. Financial incentives correlated weakly with medication use, self-efficacy for quitting smoking, and quit success. Training program evaluation was weakly to moderately correlated with each of the psychosocial mediators (attitudes, self-efficacy, and social influence), and weakly correlated with medication use, NRT use, and quit success. Quit success was correlated most with self-efficacy for quitting and attitudes about quitting. Reward responsiveness was not significantly correlated with any of the variables.

**Table 2.** Pearson Correlations Between Financial Incentives, Mediating Variables, and Smoking Cessation

	1	2	3	4	5	6	7	8	9
1. Financial incentives	1								
2. Program evaluation	0.07	1							
3. Medication use	0.11*	0.13**	1						
4. Nicotine replacement use	−0.06	0.11*	0.34***	1					
5. Reward responsiveness	0.002	0.01	−0.71	0.08	1				
6. Attitudes about quitting	0.07	0.25***	0.02	0.06	0.02	1			
7. Self-efficacy for quitting	0.14**	0.26***	0.09	−0.06	0.05	0.38***	1		
8. Social influence on quitting	0.10	0.30***	0.04	0.04	−0.03	0.34***	0.19***	1	
9. Quit success	0.15***	0.15**	0.10*	−0.08	−0.05	0.19***	0.28***	0.14**	1

*p < .05.

**p < .01.

***p < .001.

### Two-Level Path Analysis

**Figure 1.**
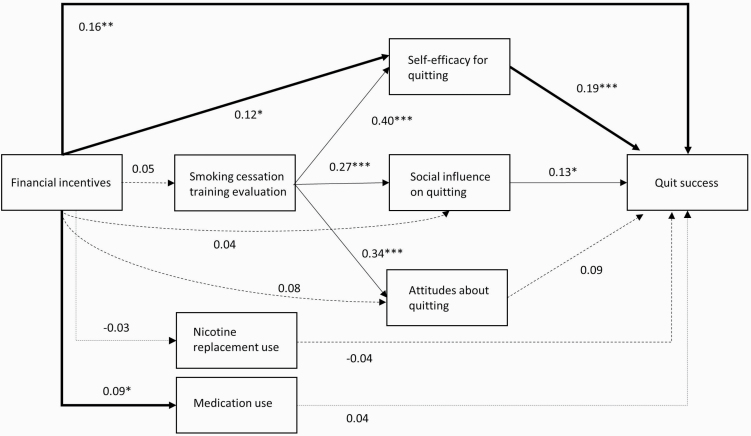
Path analysis model with unstandardized regression coefficients assessing the pathways between financial incentives and quit success. Available case analysis with multiple imputation (*n* = 604), and with random intercepts at company level. Solid arrows depict statistically significant pathways, dashed lines depict nonsignificant pathways. Thick arrows represent significant direct and mediational pathways from financial incentives to quit success. Only pathways of interest are shown. Control variables were omitted from the figure for simplification. *p < .05; **p < .01; ***p < .001.

Figure 1 presents the results of the path analysis. As supported by an average comparative fit index of 0.999, an average Tucker–Lewis index of 1.000, and an average root-mean-square error of approximation of 0.008, the model fit was good. The model explained 15.2% of the variance in quit success.

The residual intraclass correlation coefficients (ICC) indicate that there are (small) clustering effects due to persons being nested within companies: ICC = 0.089 for training program evaluation, ICC = 0.027 for attitude, ICC = 0.011 for self-efficacy, ICC = 0.051 for social influence, ICC = 0.084 for medication use, ICC = 0.068 for NRT use, and ICC = 0.037 for quit success.

The model shows that financial incentives were positively and directly associated with quit success (β = 0.16, *p* = .001). Financial incentives were also associated with a higher self-efficacy for quitting smoking (β = 0.12, *p* = .017) and with more use of smoking cessation medication (β = 0.09, *p* = .045). Financial incentives did not significantly influence appraisal of the smoking cessation program, nor influenced attitudes or social influence on quitting smoking. A higher evaluation of the smoking cessation training program was associated with a more positive attitude about quitting smoking, higher self-efficacy for quitting smoking and positive social influence on quitting smoking. Of these three psychosocial mediators, self-efficacy (β = 0.19, *p* < .001), as well as social influence (β = 0.13, *p* = .042) were associated with quit success. The complete case analysis (*n* = 328) showed comparable mediation pathways with the analysis based on multiple imputation (see Supplementary File 2). The complete case analysis did not show a significant association between financial incentives and medication use, nor an association between training program evaluation and attitude, which probably reflects a lack of statistical power due to the smaller number of participants involved in the analysis.

The associations of financial incentives with quit success and other variables in the model did not depend significantly on the participant’s reward responsiveness nor on the participant’s sex and age (all *p*-values >.15). However, the effect of the intervention on social influence and the effect on self-efficacy were close to significance moderated by educational (*p* = .051) and income level (*p* = .076), respectively. In follow-up analyses exploring these interactions, a new mediating path from financial incentives via a higher social influence (β = 0.09, *p* = .022) to a higher quit success was found for the subgroup of participants with a moderate education (but not for the low and high education groups). In addition, the association between financial incentives and self-efficacy was statistically significant for the high income group (β = 0.21, *p* = .008) and for the moderate income group (β = 0.20, *p* = .014), but was not significant for the low income group (*p* = .419). This implies that the mediating path from financial incentives via self-efficacy on quit success as previously found for the whole sample, apparently only holds for the high and moderate income groups.

## Discussion

The current study provides insight into the pathways through which financial incentives can increase quit success in smokers. In our path analysis model, financial incentives were associated with a higher use of smoking cessation medication, and incentives were associated with quit success via an indirect pathway through increased self-efficacy, but not via the smoking cessation training program evaluation, attitudes, and social influence.

The results showed a pathway from financial incentives to an increased use of smoking cessation medication. This result may be explained by the idea that financial incentives increase the determination of a smoker to be successful in this particular quit attempt, which motivates the individual to use medication in order to enhance his/her chance of quit success and earn the incentives. If incentives dependent upon quit success can motivate smokers to use smoking cessation medication, which was also found in previous studies,^16–18^ it can be an important strategy to increase successful smoking cessation^19^ that should be further explored. This result also implies that if financial incentives are used to promote smoking cessation, making cessation medication easily accessible and freely available along with the incentives may increase the intervention’s impact.^11^ Although the positive effect of smoking cessation medication use on quit success has been shown in previous research,^19^ the current study did not show a statistically significant pathway leading from medication use to quit success, likely due to a lack of statistical power.

The analysis also showed that a mediational pathway ran from financial incentives to quit success by an increased self-efficacy for quitting smoking. From previous research, it is known that there is a strong association between self-efficacy for quitting smoking and quit success,^37,38^ which is also reflected in the current model. However, this is the first study that has investigated the association between financial incentives and self-efficacy. A potential explanation of the association between the incentives and self-efficacy is that the incentives encourage people to visualize achieving their goal of quitting smoking and receiving the reward. This visualization of successfully performing a behavior (such as quitting smoking) can enhance a person’s self-efficacy for that behavior.^39^ Because a high self-efficacy has shown to be a good predictor of successful behavioral change,^37,38,40^ financial incentives may be a novel way to increase self-efficacy and improve quit success which should be explored in further research. Another possibility for the association between the incentives and self-efficacy, is the self-efficacy-as-motivation argument,^41^ which states that because of the way that self-efficacy is measured, by asking how confident a person is to perform or resist a certain behavior, self-efficacy scores reflect motivation.^41^ This implies that the positive association in the current study between the incentives and self-efficacy actually represents an increase in the participants’ motivation to quit smoking elicited by the financial incentives. Remarkably, the mediational pathway through self-efficacy only seemed to apply to the high and moderate income groups, but not the low income group. This finding should be replicated and explored further in future research.

The main results of the current study did not show significant pathways between financial incentives and quit success via attitudes and social influence. It is possible that because the average attitude toward quitting was already very positive in the current sample (mean score 4.7 out of 5.0), the incentives could not further increase this positive attitude. It should be further assessed whether in populations of smokers with less positive attitudes toward smoking cessation incentives could make a difference. The follow-up analyses did show a mediational pathway from financial incentives via a higher social influence to quit success, but only for the subgroup of participants with a moderate education level. Future research should explore whether this education-dependent association between financial incentives and social influence for quitting smoking can be replicated and whether and why it is dependent on educational level. Previous research suggests that financial incentives may be even more effective in stimulating support from the smoker’s social environment if they are shared with significant others, for example as was done in a study with pregnant women^23^ where both the woman and a designated social supporter received financial incentives if the expectant mother quit smoking.

The direct effect of financial incentives that was revealed in the current model may indicate that incentives work separately from the established pathways leading to behavioral change. It is also possible that this pathway is mediated by other mediators like intention or action planning^42^ which we did not measure in the current study.

A path that was not statistically significant in the current study, was the path leading from financial incentives via smoking cessation training and the psychosocial mediators to quit success. The absence of an association between the financial incentives and training appraisal may indicate that financial incentives do not affect how participants value a smoking cessation training program. Alternatively, the high appreciation of the training by the participants may have caused a ceiling effect. Nonetheless, the model indicated that the evaluation of the group smoking cessation training program was strongly associated with social influence, self-efficacy, and attitudes. This result was not surprising, since the group training program included components aiming to increase these psychosocial mediators, and since group behavioral therapy has proven to be an effective method for smoking cessation.^43^ The results of the current study demonstrate an important contribution of the smoking cessation training program to predictors of quit success, and we therefore recommend that financial incentives should be used in combination with an effective behavioral intervention.

Contrary to our hypothesis, we did not find that individual reward responsiveness affected the association between financial incentives and quit success. It is possible that, because we advertised during the recruitment period that there was a chance to receive financial incentives, the participants who subscribed to our study mainly consisted of people with a high reward responsiveness, which could have resulted in selection bias. Yet, the mean scale score of 3.6 found in the current study is comparable to scores found in previous research ranging from 3.24 to 3.52^30,44–46^ and thus may not be particularly high. However, it may be possible that our participants all exceeded a certain threshold level of reward responsiveness, above which the incentives were not increasingly effective to stimulate quit success. Alternatively, the BAS Reward Responsiveness Scale may measure a more general trait and might not be sensitive enough to measure differences in responsiveness to the financial incentives. There may be other effect modifiers, such as individual differences in impulsiveness or the preference for immediate versus delayed rewards (delay discounting),^5^ which could be explored in further research.

### Strengths and Limitations

This is the first study that investigates the causal pathways leading from providing financial incentives for smoking cessation to quit success. Important strengths of this study are that the data are from a CRT, which increases confidence in the causal effect of financial incentives on quit success because of the randomization into intervention and control groups, and the biochemical validation of smoking abstinence. A limitation of the design of the current study is that it does not allow interpreting all associations as causal effects. In the current study, we used a cutoff value of 9 ppm based on West et al.^31^ While it has been suggested that a cutoff criterion of less than or equal to 4 ppm is more sensitive to detect recent smoking,^47^ using this stricter criterion would not have changed our conclusions as only seven of the 206 abstinent participants had a CO value above 4 ppm.

Another limitation of the current study is that we could not incorporate the psychosocial mediator “intention to quit smoking,” in the model, which according to behavioral change theories^13,14,21^ is predicted by attitudes, social influence, and self-efficacy, and precedes behavior. The reason that we could not include intention to quit in the model is that at the follow-up measurement directly after finishing the smoking cessation training program, our study population included both smokers and successful quitters, and only participants who had not successfully quit smoking were asked whether they intended to quit smoking in the (near) future. It is possible that by not being able to include intention to quit in the model, we have missed a mediator of the effect of financial incentives. Another issue is that the scale for measuring “social influence” had a Cronbach’s alpha of 0.62, which is below, what is considered to be an acceptable level of 0.70.^48^ However, the construct social influence was considered a formative construct in the current study, and therefore its internal consistency is of little importance.^49^ The scale for measuring “attitude” had a Cronbach’s alpha of 0.67, also being below the acceptable level of 0.7, which may have led to attenuation effects in the analysis. A factor that should be taken into consideration is that participants in the current study were probably intrinsically motivated to quit smoking, because they voluntarily signed up for an extensive smoking cessation program without the certainty of receiving financial incentives. Therefore, the paths found in the current study may not be generalizable to extrinsically motivated smokers.

## Conclusions

This study provides insight in how financial incentives increase quit success, and our findings suggest that financial incentives may be used to increase medication use and self-efficacy for quitting smoking, which offers an indirect way to improve successful smoking cessation.

## Supplementary Material

A Contributorship Form detailing each author’s specific involvement with this content, as well as any supplementary data, are available online at https://academic.oup.com/ntr.

ntaa024_suppl_Supplementary_File_1

ntaa024_suppl_Supplementary_File_2

ntaa024_suppl_Supplementary_Taxonomy_Form
