# Supplementary material for: How Financial Incentives Increase Smoking Cessation: A Two-Level Path Analysis
Source: Nicotine Tob Res. 2020 Jan 29;23(1):99–106. doi: 10.1093/ntr/ntaa024 (PMC7789935; doi:10.1093/ntr/ntaa024)
Supplement: ntaa024_suppl_Supplementary_File_1 [file ntaa024_suppl_supplementary_file_1.pdf]

Supplementary file 1: Questions to evaluate the smoking cessation training programme

The following statements are about the stop-smoking group training. Indicate to what extent you agree with the following statements:

Response categories:
(1) Completely disagree (2) Disagree (3) Don't disagree/don't agree (4) Agree (5) Completely agree (6) Don't know
* Items f and g were reverse scored.

a. I found the program interesting

b. I found the program educational

c. I found the program clear

d. I liked the program

e. I have received sufficient information about quitting smoking

f. Participating in the program took me a lot of effort*

g. Participating in the program took a lot of time*

h. I thought the total duration of the program was good

i. The program has met my needs

j. I will recommend participating in the training if someone asks me to do so

k. I am satisfied with the quality of the program

l. I am satisfied with the type of help I have received through the program

m. Communication between my coach and me during the program went well

What rating would you give the stop-smoking group training?

1 2 3 4 5 6 7 8 9 10
