# Supplementary material for: How Financial Incentives Increase Smoking Cessation: A Two-Level Path Analysis
Source: Nicotine Tob Res. 2020 Jan 29;23(1):99–106. doi: 10.1093/ntr/ntaa024 (PMC7789935; doi:10.1093/ntr/ntaa024)
Supplement: ntaa024_suppl_Supplementary_File_2 [file ntaa024_suppl_supplementary_file_2.pdf]

Supplementary file 2. Path analysis model with unstandardized regression coefficients assessing the pathways between financial incentives and quit success.

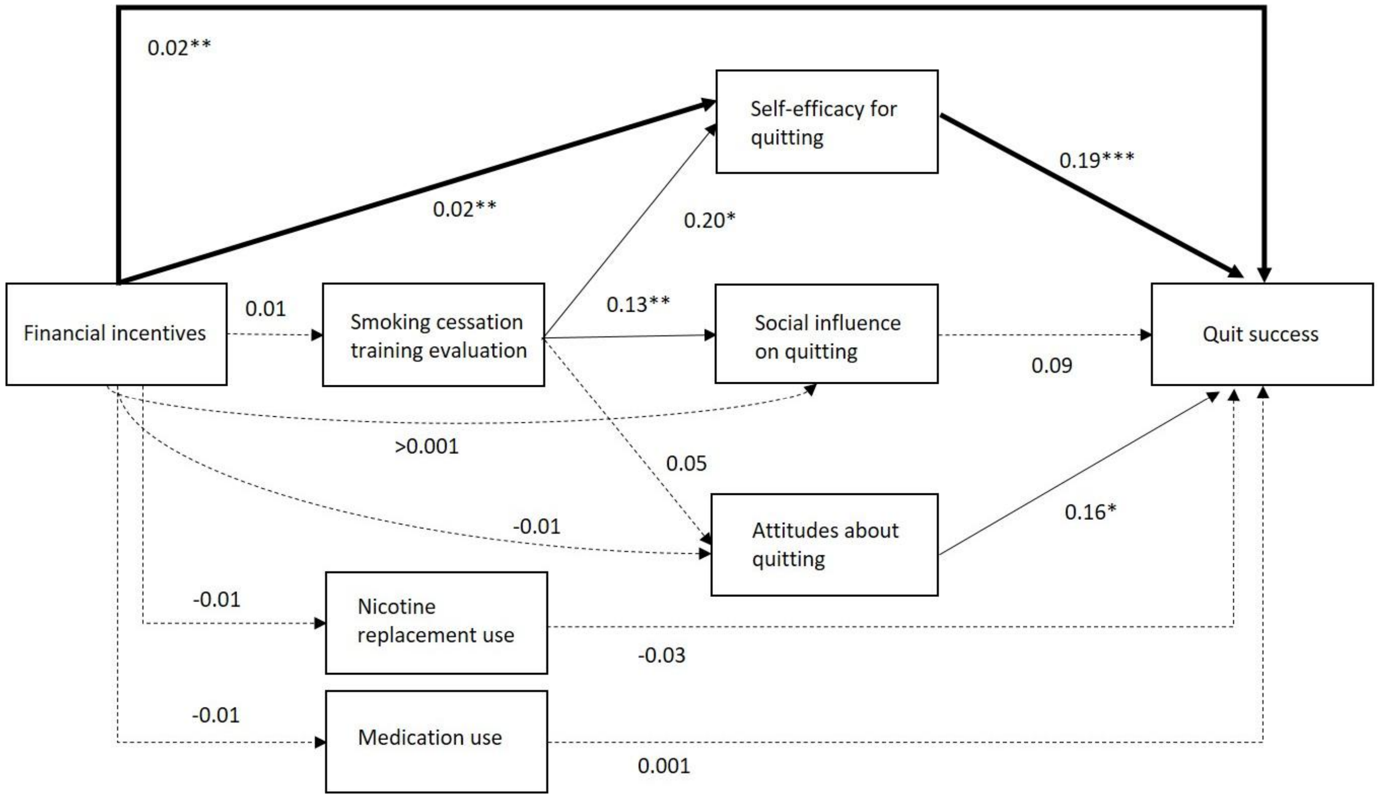

Complete case analysis (n=328) with random intercepts at company level. Solid arrows depict statistically significant pathways, dashed lines depict non-significant pathways. Thick arrows represent significant direct and mediational pathways from financial incentives to quit success. Only pathways of interest are shown. Control variables were omitted from the figure for simplification.
